# Clonal Wars: Monoclonal Antibodies Against Infectious Pathogens

**DOI:** 10.1089/dna.2021.0457

**Published:** 2022-01-12

**Authors:** Joshua Tan

**Affiliations:** Antibody Biology Unit, Laboratory of Immunogenetics, National Institute of Allergy and Infectious Diseases, National Institutes of Health, Rockville, Maryland, USA.

**Keywords:** monoclonal antibodies, SARS-CoV-2, infectious disease

## Abstract

Monoclonal antibodies are coming of age as powerful tools for the prevention of infectious diseases. In recent years, the rate of antibody discovery has accelerated, and the coronavirus disease 2019 (COVID-19) pandemic has shone a spotlight on the role of these antibodies in combating pathogens. However, questions remain about the utility of monoclonal antibodies, especially when effective vaccines are also available. In this article, I discuss the role of monoclonal antibodies and briefly describe the effort to identify potent human monoclonal antibodies against severe acute respiratory syndrome coronavirus 2 (SARS-CoV-2), including our study on bispecific antibodies that neutralize SARS-CoV-2 variants of concern.

## Introduction

The human body is continuously exposed to microorganisms, some of which have the potential to inflict fatal harm. Our immune systems have the complex task of protecting the body from a bewildering array of pathogens that differ greatly at the molecular level and elude a one-size-fits-all response. Antibodies are large glycoproteins produced by B cells that are key agents in this defense. Antibodies act as homing missiles and attach to specific parts of invading microorganisms through a lock-and-key mechanism, disrupting their activity and often leading to their destruction.

Owing to the unique developmental pathway of B cells, each cell produces a slightly different antibody (or “key”) with a unique conformation. Therefore, B cells as a population have the potential to produce an enormous variety of antibodies, which are diverse enough to handle virtually any invading pathogen, regardless of the conformation of the molecules (“locks”) on its surface. Collections of antibodies from a population of B cells, such as antibodies in serum samples, are referred to as polyclonal antibodies. Conversely, an antibody derived from a single B cell clone is called a monoclonal antibody, and thus monoclonal antibody research is the study of single antibodies at the molecular level.

## Why Study Monoclonal Antibodies?

**FIG. 1.**
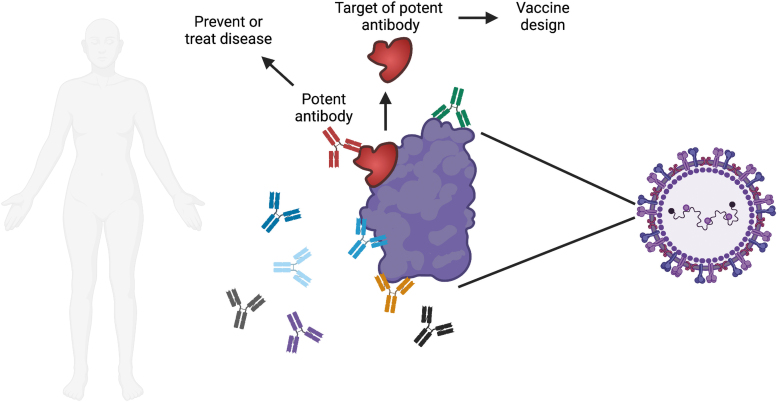
Overview of monoclonal antibody research. Each person makes many different antibodies in response to infection or vaccination. One major goal of monoclonal antibody research is to identify the most potent antibodies against a specific pathogen of interest. Potent antibodies can be tested for the ability to prevent or treat disease in humans. The site on the pathogen that is bound by the potent antibody can be evaluated for use in a vaccine, with the goal of triggering the production of these potent antibodies when the vaccine is administered. Image created with Biorender.com.

One major goal of monoclonal antibody research is to identify the most potent monoclonal antibodies that target a pathogen of interest (Fig. 1). This can be quite a treasure hunt, since not all antibodies that target a pathogen work equally well in eliminating the pathogen. Some may stick too weakly to the pathogen to have an effect, whereas others may attach to a region that has no function and, therefore, do not inconvenience the pathogen.

Other antibodies can even act as double agents and help the pathogen by acting as a bridge between a pathogen and a human cell that the pathogen wants to invade. In contrast, potent monoclonal antibodies typically act by binding strongly to a part of the pathogen that is required for its function, stopping it from invading cells or calling over allies from other sectors of the immune system to destroy the microorganism. Finding these potent antibodies is useful for two reasons.

First, they can be used to identify the part of the pathogen (usually a part of a protein or carbohydrate) that is the most susceptible to antibody attack. This so-called epitope can then be investigated as a vaccine candidate that triggers a focused potent antibody response against that pathogen. Second, potent antibodies can be tested directly for their ability to prevent or treat disease. This has already been used successfully in several cases—for instance, the monoclonal antibody palivizumab has been used for >20 years to prevent respiratory syncytial virus infection in high-risk infants (Olchanski *et al.*, 2018).

## With Vaccines Around, Do Monoclonal Antibodies Matter?

Under most circumstances, vaccines that work well are the most effective way to prevent infectious disease, as is being seen in the coronavirus disease 2019 (COVID-19) pandemic (Tregoning *et al.*, 2021). Successful vaccines work by preparing the immune system to eliminate a given pathogen, most often by triggering the production of potent antibodies against that pathogen. Hence, when the pathogen arrives, it finds an army of antibodies (and immune cells) ready to take it down before it can gain a foothold and spread in the body. One major function of monoclonal antibodies, such as vaccines, is to act as a prophylactic agent to prevent disease. Therefore, if a good vaccine is already available for a given disease, is it still useful to search for monoclonal antibodies?

I think so, for several reasons. Monoclonal antibodies are not just alternatives to vaccines, but are also tools that can improve existing vaccines by marking specific sites of pathogen vulnerability, as already described. Moreover, monoclonal antibodies have properties that make them a useful alternative to vaccines in specific cases. First, antibodies provide immediate protection, whereas vaccines take longer (often several weeks) to provide full protection.

Thus, antibodies can be administered in outbreak settings when there is not enough time to wait for a vaccine to work. Second, vaccines rely on a strong host immune response to be effective and may not work well in certain groups of individuals, such as the immunocompromised, including hospitalized patients who take immunosuppressive medication (Boyarsky *et al.*, 2021). In contrast, monoclonal antibodies can attack the pathogen directly and do not usually require a strong host immune system.

Third, monoclonal antibodies can be used for treatment, particularly for early stage disease, whereas vaccines only work by preventing disease. A striking example of this application is a recent report that early stage Ebola patients given the monoclonal antibody mAb114 or a cocktail of three antibodies called REGN-EB3 had a ∼90% survival rate (Maxmen, 2019). Finally, there are diseases for which we do not yet have effective vaccines, such as AIDS. Here, potent monoclonal antibodies could be used to prevent infection and disease while the search for an effective vaccine continues. Indeed, clinical trials with monoclonal antibodies against HIV-1 are currently being performed to test their efficacy (Crowell *et al.*, 2019).

## Drawbacks of Monoclonal Antibodies and Potential Solutions

An oft-cited disadvantage of using monoclonal antibodies to prevent infectious disease is their high cost, as the pipeline needed to produce monoclonal antibodies at a quality that is suitable for use in humans is complex and expensive. However, efforts by key stakeholders and continuously improving technology are reducing this cost to a level wherein it is starting to appear feasible for large-scale administration. The use of alternative technologies such as nanobodies, as well as adeno-associated virus or mRNA platforms to deliver antibodies, may also be more cost-effective (Martinez-Navio *et al.*, 2019; Van Hoecke and Roose, 2019; Jovčevska and Muyldermans, 2020).

Moreover, the discovery of monoclonal antibodies with ever increasing potency will help in this area, since these can be given at a lower and thus cheaper dose. Another potential drawback is that monoclonal antibodies are not self-sustaining, whereas vaccines usually induce a long-lasting protective immune response. Thus, a monoclonal antibody given to an individual would eventually be broken down, and continuous protection would require regular redosing. Recent advances in antibody engineering have increased the longevity of antibodies through modifications of the “stem” of the antibody (Zalevsky *et al.*, 2010).

This extended longevity has opened the doors to new applications. For instance, a potent monoclonal antibody with extended longevity could be used to prevent malaria in regions where transmission predictably occurs during a defined rainy season each year, such as in the Sahel region of sub-Saharan Africa. In addition, monoclonal antibodies, by nature, target one specific site on the pathogen. This makes them susceptible to mutations in the microorganism that change the shape of the target site such that the antibodies can no longer attach. To overcome this issue, monoclonal antibodies should be selected based on target sites that tend not to change across different strains of the same pathogen (Corti *et al.*, 2021).

Moreover, a cocktail of multiple monoclonal antibodies that target different regions or the use of bispecific antibodies that combine multiple monoclonal antibodies into a single molecule can alleviate this issue (Cho *et al.*, 2021). Finally, the possibility of antibody-dependent enhancement, where antibodies make disease worse instead of stopping disease, must be considered. This issue is related to antibodies that link pathogens and specific target cells, thus increasing the rate of invasion of these cells, or to antibodies that cause the immune system to overreact in a harmful way. This can be monitored to ensure that antibodies with this feature are not taken forward for clinical development.

## A Case Study: Monoclonal Antibodies to Treat COVID-19

An example that connects many of the points already discussed is the use of monoclonal antibodies during the COVID-19 pandemic. This pandemic has brought home the devastation wrought by an uncontrolled infectious agent. Since the beginning of the pandemic, an intense effort by different research laboratories, including my own, has been made to identify potent monoclonal antibodies against severe acute respiratory syndrome coronavirus 2 (SARS-CoV-2) (Liu *et al.*, 2020; Robbiani *et al.*, 2020; Rogers *et al.*, 2020; Zost *et al.*, 2021; Cho *et al.*, 2021). This effort has been viewed as a complementary approach to vaccines and has continued in full force even after several COVID-19 vaccines were shown to be highly effective.

The research community has now generated a large number of potent neutralizing antibodies against SARS-CoV-2 at record speed, including three products that have been authorized by the U.S. Food and Drug Administration for treatment of mild-to-moderate COVID-19 patients who are at risk of progressing to more severe disease (Weinreich *et al.*, 2020; Gottlieb *et al.*, 2021; Gupta *et al.*, 2021).

These antibodies have been reported to be effective in reducing COVID-19–related hospitalization and deaths (Taylor *et al.*, 2021). Monoclonal antibodies may also be useful to downmodulate a pathogenic immune response during the course of an infectious disease. For example, the antibody tocilizumab, which targets the immune protein IL-6, is currently being used to treat specific cases of severe COVID-19 (Gupta and Leaf, 2021).

A major concern has been whether these monoclonal antibodies are still effective against the variants of concern that have emerged in the past year. To address this concern, our group has generated bispecific antibodies that target different regions of the SARS-CoV-2 spike protein, the main target on the surface of the virus (Cho *et al.*, 2021). Several of these antibodies have been shown to be highly effective in neutralizing SARS-CoV-2 in cell culture experiments and in preventing disease in an animal model.

Since these bispecific antibodies target two different sites on the spike protein, they will be less affected by mutations that change the shape of a single site, and indeed two of these antibodies were shown to be fully functional against the alpha, beta, gamma, and delta variants. As new variants continually emerge, we and the wider research community will continue testing the available pool of antibodies to ensure that they remain effective against these variants.

## Future Perspectives

The 21st century has already witnessed the emergence of three deadly coronaviruses, an influenza pandemic, and several Ebola outbreaks. Unfortunately, this trend is likely to continue with increased interactions with wildlife harboring undiscovered pathogens and the expansion of global travel that facilitates rapid spread of new pathogens. The research community should be ready not only to respond, but also to prepare for the emergence of new pathogens. Monoclonal antibody discovery can help in this area. For example, a potent monoclonal antibody that cross-reacts with multiple coronaviruses would be very useful in preventing future coronavirus outbreaks. Therefore, monoclonal antibodies are likely to continue to be powerful tools in the war against infectious diseases.
